# A Rare Case of Watery Vaginal Discharge due to Caesarean Scar Dehiscence following Brace Suture and Balloon Tamponade for the Management of Postpartum Hemorrhage

**DOI:** 10.1155/2020/2064782

**Published:** 2020-02-26

**Authors:** Krystal Koh, Devendra Kanagalingam, Rajeswari Kathirvel

**Affiliations:** ^1^Department of Obstetrics and Gynecology, KK Women's and Children's Hospital, Singapore; ^2^Department of Obstetrics and Gynecology, Singapore General Hospital, Singapore; ^3^Department of Obstetrics and Gynecology, KK Women's and Children's Hospital, Lee Kong Chian School of Medicine, Duke NUS Medical School and Yong Loo Lin School of Medicine, Singapore

## Abstract

A woman in her early twenties with dichorionic diamniotic twins underwent emergency caesarean section (CS) for failed induction of labor for discordant growth at 37 weeks. Her CS was complicated by atonic postpartum hemorrhage (PPH) requiring uterotonics, B-lynch suture, and Bakri balloon. She presented on the 5^th^ postoperative day (POD) with fever and wound pain and collapsed due to desaturation. Investigations confirmed ascites on computed tomography (CT) of her abdomen and cardiomyopathy on echocardiogram. She was readmitted on the 22^nd^ POD with watery vaginal discharge. CT abdomen revealed a dehisced CS scar and loculated ascites. Her discharge settled after three weeks with antibiotics and drainage of the ascites. A CT scan 3 months later showed reduction of the peritoneal collection. Caesarean scar dehiscence should be considered for patients presenting with ascites and vaginal discharge after a CS, particularly in the presence of risk factors such as infection or anemia.

## 1. Introduction

Intra-abdominal infection (IAI) and peritonitis are known complications of caesarean section (CS). Endomyometritis, uterine incisional necrosis and dehiscence, and perioperative bladder and/or bowel injury are possible causes [1]. Uterine dehiscence occurs in 0.06 to 3.8% of caesarean cases [2]. Risk factors include diabetes, emergency surgery, infection, suture technique, hematoma on the uterine incision line, and retrovesical hematoma [3]. While peritonitis often presents as abdominal pain and fever, the presence of vaginal discharge should raise suspicion of uterine dehiscence or a vesicovaginal fistula. We present an unusual case of watery vaginal discharge in a young woman post-CS that was complicated by postpartum hemorrhage (PPH) managed with brace suture and balloon tamponade.

## 2. Case Presentation

A woman in her early twenties with dichorionic diamniotic twins, antenatally noted to have iron deficiency anemia, underwent an emergency caesarean section (CS) following failed induction of labor for discordant growth of twins at 37 weeks gestation. Her CS was complicated by atonic PPH of 1.2 liters (L) that was managed by uterotonics, B-lynch suture, and insertion of a Bakri balloon. Postoperatively, her hematocrit was 20.3% and hemoglobin was 6.4 g/dL, from a preoperative hematocrit and hemoglobin of 29.7% and 9.0 g/dL, respectively. She received 4 units of packed cell transfusion postoperatively followed by oral hematinics and was discharged on the 3^rd^ POD in a stable condition with a hematocrit of 24.6% and hemoglobin of 8 g/dL.

**Figure 1:**
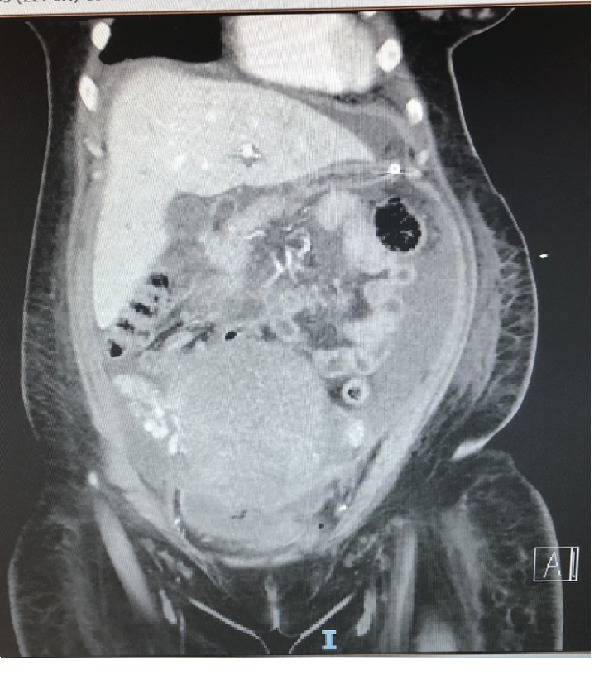
CT abdomen and pelvis showing ascites out of keeping with recent caesarean section.

She was readmitted on the 5^th^ POD with fever, wound site pain, and blood pressure of 144/89 mm of Hg. On readmission, her hemoglobin was 7.8 g/dL with hematocrit of 24.5%, total white cell count was normal at 8.29 × 10^9^/L, and urinalysis showed a predominance of white cells. She was treated empirically for urinary tract infection and given oral labetalol for her hypertension. She collapsed the next day on the ward due to desaturation (SpO_2_ 85% on nonrebreather mask) associated with hypertension of 191/70 mm of Hg and tachycardia of 180 to 190 beats per minute (bpm). She was stabilized in the intensive care unit (ICU) with endotracheal intubation, adenosine for supraventricular tachycardia, and intravenous magnesium sulfate for presumed preeclampsia, followed by noradrenaline for subsequent hypotension due to septic shock. Initial differentials included preeclampsia, pulmonary embolism, type 2 myocardial infarction, and pneumonia complicated by septic shock. She was thereafter thoroughly investigated, and the positive findings were that of a cardiomyopathy confirmed by echocardiography with a left ventricular ejection fraction of 25 to 30% and global hypokinesia, ascites confirmed by computed tomography (CT) of her abdomen and pelvis (Figure 1), and positive blood cultures for *Bacteroides fragilis*. Her cardiomyopathy was thought to be precipitated by anemia and *Bacteroides fragilis* bacteremia from a likely pelvic source. She was transferred to a coronary care unit and recovered clinically with supportive treatment for cardiomyopathy and broad spectrum antibiotics. She received a total of 7 days of intravenous (IV) piperacillin/tazobactam and 9 days of metronidazole, oralized to amoxicillin/clavulanic acid for 2 weeks on discharge. She was started on perindopril, bisoprolol, spironolactone, and ivabradine for optimization of her cardiac function. She was discharged on the 16^th^ POD in a stable condition.

**Figure 2:**
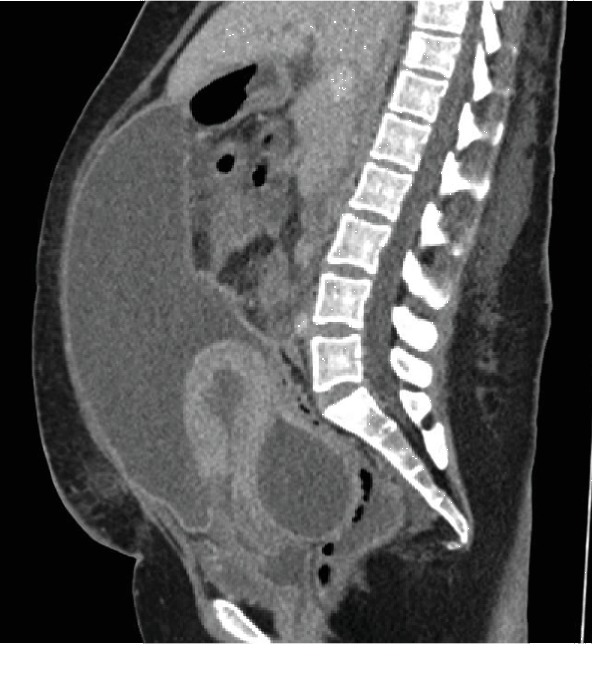
Initial CT findings of a dehisced CS scar and increase in size of the loculated ascites.

She was readmitted on the 22^nd^ POD with copious amounts of watery vaginal discharge. The initial impression was that of a vesicovaginal fistula. A CT scan of the abdomen and pelvis ruled out a urinary tract injury but confirmed a dehisced CS scar and loculated ascites with the possibility of superimposed infection. The ascites increased in size from the previous CT scan (Figure 2). She was treated with antibiotics, and the ascites was drained abdominally under ultrasound guidance. She received 13 days of IV ceftriaxone and metronidazole, oralized to ciprofloxacin and metronidazole for another 2 weeks. A total of 850 mL of hemoserous fluid was drained from the abdomen within 24 hours of the drain insertion. The total fluid drainage over 13 days was 3560 mL. Ascitic fluid cultures yielded no bacterial or fungal growth. Vaginal swab and vaginal discharge cultures revealed mixed flora.

**Figure 3:**
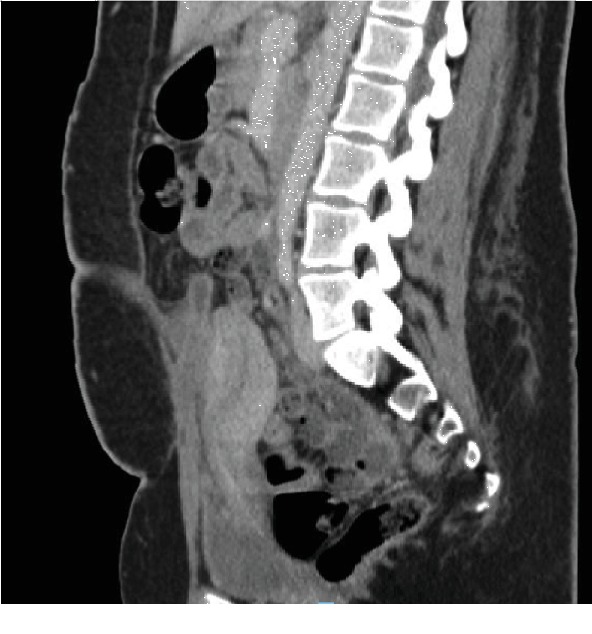
Follow-up CT scan showing reduction of the abdominal fluid collection after drainage and antibiotics 3 months after drainage (4 months postoperatively).

Interval scans showed a marked reduction in the fluid collection, and she was discharged two weeks later on the 36^th^ POD. Her vaginal discharge eventually settled after three weeks. After discharge, she was reviewed in the outpatient clinic, and interval ultrasound scans of the pelvis one and two months later and a CT scan three months later showed further reduction of the pelvic collection with only a sliver of fluid in the lower abdomen at the fourth month postoperatively (Figure 3). She was thereafter discharged from the gynecology clinic. She was also seen in the cardiology outpatient clinic two months after discharge and was stable. Unfortunately, she defaulted on subsequent cardiology follow-up visits for a repeat echocardiogram.

**Figure 4:**
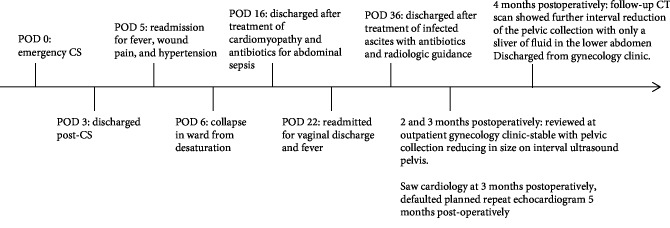
Timeline of patient's course.

A summary of the patient's clinical course is shown in Figure 4.

## 3. Discussion

This patient had vaginal discharge due to leaking of inflammatory exudate following IAI through the dehisced CS wound. It is rather unusual to see ascitic fluid draining through the dehisced CS scar into the vagina. Plausible pathophysiologies include uterine endomyometritis predisposing to uterine scar dehiscence leading to secondary peritonitis and ascites, primary peritonitis predisposing to dehiscence of the uterine scar and drainage of ascites through the vagina, and inflammatory ascites as a result of discharge of lochia into the abdominal cavity via the dehisced uterine wound, with resultant negative ascitic fluid culture.

Our patient required concurrent use of brace suture and balloon tamponade (sometimes called the “uterine sandwich” technique) [4] to manage her postpartum hemorrhage. Sepsis following this could have contributed to the uterine dehiscence that in turn could have led to peritonitis. Cardiomyopathy and anemia compromising perfusion could have been additional risk factors for uterine dehiscence in this patient. Other predisposing factors for peritonitis include immunocompromise, genital infection [5], and inadvertent bladder and/or bowel injury. These were not seen in this case. Uterine necrosis following the “uterine sandwich” technique has been described [6, 7] and could also have led to dehiscence; however, this was thought to be less likely as she was clinically well at the time of second admission to our hospital.

Cases of postcaesarean peritonitis in the literature describe uncommon infective organisms including *Candida* [8] and *Streptococcus pneumoniae* [9], along with noninfective etiologies such as reactive [10] and vernix caseosa [11] peritonitis. Similar cases of uterine dehiscence complicated by peritonitis have varied clinical presentations including fever, abdominal pain, distension, vaginal discharge, wound infection [12], and PPH [13]. These features manifested from anywhere between the first POD to two months postoperatively. Our patient similarly presented with fever and wound site pain on the 5^th^ POD and watery vaginal discharge with fever on the 22^nd^ POD.

A broad range of differential diagnoses was considered for each of the presenting complaints for this patient in view of nonspecific features at presentation. On initial presentation when she presented on the 5^th^ POD with fever, wound site pain, and headache with a temperature of 38.3 degrees Celsius and blood pressure of 144/89 mm of Hg, the initial differentials included fever secondary to urinary tract infection and possible preeclampsia. Following her collapse on 6^th^ POD due to desaturation, hypertension, and tachycardia, further investigations revealed raised serum troponin T, and a chest X-ray and CT pulmonary angiogram showed bilateral lower lobe consolidation with diffuse patchy ground glass opacities of both lungs, and CT of the abdomen and pelvis revealed ascites that were more than what would have been expected with the recent caesarean section. She also subsequently developed hypotension with systolic blood pressure dropping to a nadir of 70 mm of Hg with a temperature of 38 degrees Celsius, attributed to septic shock. Differentials at this point were acute pulmonary oedema secondary to cardiomyopathy and sepsis from respiratory, urinary, and abdominal sources. Postpartum endometritis, infected intra-abdominal collection, urinary tract infection, and pelvic septic thrombophlebitis were considered as a possible source for pelvic sepsis, and vesicovaginal fistula was considered as a differential for the watery vaginal discharge when she was readmitted on the 22^nd^ POD.

Stepwise investigations are crucial in such cases and should include serum inflammatory markers, microbiology (blood culture, urine culture, ascitic fluid culture, vaginal swab, and vaginal fluid culture), and serial ultrasound and CT scans, which were performed on this patient.

Management options for uterine dehiscence include conservative treatment with antibiotics and drainage. However, in the presence of infection, laparotomy with either repair of the uterine defect or hysterectomy should be considered [1, 3, 14]. Fortunately, our patient settled with conservative management with antibiotics and drainage.

As there are varied etiologies and unusual presentations of intra-abdominal infection postcaesarean, there should be a high index of suspicion for uterine dehiscence and bladder or bowel injury with any postoperative fluid accumulation or abnormal vaginal discharge postoperatively.

This case also demonstrates multiple complications occurring simultaneously in the postpartum period including PPH, cardiomyopathy precipitated by anemia and sepsis, and uterine dehiscence. Optimal management by a multidisciplinary team consisting of obstetricians, anesthetists, cardiologists, respiratory medicine, and infectious disease specialists eventually led to a good outcome.
